# Assessment of musculoskeletal abnormalities in children with mucopolysaccharidoses using pGALS

**DOI:** 10.1186/1546-0096-12-32

**Published:** 2014-08-01

**Authors:** Mercedes O Chan, Ethan S Sen, Elizabeth Hardy, Pauline Hensman, Edmond Wraith, Simon Jones, Tim Rapley, Helen E Foster

**Affiliations:** 1 Paediatric Rheumatology, Institute of Cellular Medicine, The Medical School, Framlington Place, Newcastle University, Newcastle upon Tyne NE2 4HH, UK; 2 Paediatric Rheumatology, Great North Children’s Hospital, Royal Victoria Infirmary, Newcastle upon Tyne NHS Hospitals Foundation Trust, Queen Victoria Road, Newcastle upon Tyne NE1 4LP, UK; 3 Willink Biochemicals Genetics Unit, Royal Manchester Children’s Hospital, Central Manchester University Hospitals NHS Foundation Trust, Oxford Road, Manchester M13 9WL, UK; 4 Institute of Health and Society, Newcastle University, Baddiley Clark Building, Richardson Road, Newcastle upon Tyne NE2 4AX, UK; 5 Division of Paediatric Rheumatology, Department of Paediatrics, BC Children's Hospital and the University of British Columbia, K4-119 Ambulatory Care Building, 4480 Oak Street, Vancouver BC V6H 3V4, Canada

**Keywords:** pGALS, Mucopolysaccharidoses, Musculoskeletal examination, Metabolic disease

## Abstract

**Background:**

Children with mucopolysaccharidoses (MPS) often have musculoskeletal (MSK) abnormalities. Paediatric Gait, Arms, Legs, and Spine (pGALS), is a simple MSK assessment validated in school-age children to detect abnormal joints. We aimed to identify MSK abnormalities in children with MPS performing pGALS.

**Methods:**

Videos of children with a spectrum of MPS performing pGALS were analysed. A piloted proforma to record abnormalities for each pGALS manoeuvre observed in the videos (scored as normal/abnormal/not assessable) was used by three observers blinded to MPS subtype. Videos were scored independently and rescored for intra- and inter-observer consistency. Data were pooled and analysed.

**Results:**

Eighteen videos of children [12 boys, 6 girls, median age 11 years (4–19)] with MPS (13 type I [5 Hurler, 8 attenuated type I]; 4 type II; 1 mannosidosis) were assessed. The most common abnormalities detected using pGALS were restrictions of the shoulder, elbow, wrist, jaw (>75% cases), and fingers (2/3 cases). Mean intra-observer Κ 0.74 (range 0.65–0.88) and inter-observer Κ 0.62 (range 0.51–0.77). Hip manoeuvres were not clearly demonstrated in the videos.

**Conclusions:**

In this observational study, pGALS identifies MSK abnormalities in children with MPS. Restricted joint movement (especially upper limb) was a consistent finding. Future work includes pGALS assessment of the hip and testing pGALS in further children with attenuated MPS type I. The use of pGALS and awareness of patterns of joint involvement may be a useful adjunct to facilitate earlier recognition of these rare conditions and ultimately access to specialist care.

## Background

The mucopolysaccharidoses (MPS) are a group of rare inherited metabolic disorders with a spectrum of phenotypes resulting from glycosaminoglycans accumulating in cells due to deficiencies in the enzymes required for their catabolism [1]. MPS are progressive multisystem disorders ranging from attenuated to severe depending on the degree of enzyme inactivity (Table 1). Enzyme replacement therapy (ERT) and haematopoietic stem cell transplant (HSCT) are available for some types of MPS, which may improve physical features, cognition and function [2-4].

**Table 1:** Overview of the mucopolysaccharidoses (MPS)

**MPS Type**	**Main clinical features**	**Enzyme deficient**	**Substrate accumulated**
**Skeletal disease, soft tissue storage and a range of central nervous system disease**			
**I-H** Hurler	Developmental delay, coarse facial features, dysostosis multiplex, hepatosplenomegaly, death by age 10 years	Alpha-L-iduronidase	Heparan sulfate
**I-HS** Hurler-Scheie	Micrognathia, toe walking, moderate coarse facies, possible normal intelligence, death by 20s (phenotype intermediate between I-H and I-HS)		Dermatan sulfate
**I-S** Scheie	Aortic valve disease, joint disease, corneal clouding, normal facies, survive into adulthood		
**II** Hunter	Severe: Disease course similar to MPS I-H, but clear corneas, aggressive behaviour and developmental delay	Iduronate sulfatase	Heparan sulfate
	Mild: Normal or near-normal intelligence, less pronounced physical features		Dermatan sulfate
**MPS VII** Sly	Variable intermediate presentation similar to MPS I, from fetal hydrops to mild dysmorphism; dense inclusions in granulocytes	Beta-glucuronidase	Heparan sulfate
			Dermatan sulfate
**MPS VI** Maroteaux-Lamy	Similar to MPS I without CNS disease, pachymeningitis cervicalis, death in teens and 20s; Hurler phenotype with marked corneal clouding; mild, moderate and severe expression in different families	N-acetylgalactosamine-4-sulfatase (arylsulfatase B)	Dermatan sulfate
**Skeletal, cartilage and ligament disease primarily**			
**MPS IVA** Morquio A	Skeletal disease (bone dysplasia) with short stature, ligamentous laxity, corneal opacities, final height <125 cm	N-acetylgalactosamine-6-sulfatase	Keratan sulfate
**MPS IVB** Morquio B	Same as IV-A but milder; adult height >120 cm	Beta-galactosidase	
**MPS IX** Natowicz syndrome	Periarticular masses, nodular synovium, popliteal cyst, large joint effusion	Hyaluronidase 1	Hyaluronan
**MPS IIIA** Sanfilippo A	Behavioural problems, sleeping disorder, aggression, progressive dementia, mild dysmorphism, coarse hair, clear corneas, survival to adulthood possible	Sulfamidase	Heparan sulfate
**MPS IIIB** Sanfilippo B		Alpha-N-acteylglucosaminidase	
**MPS IIIC** Sanfilippo C		GAC-acteylase	
**MPS IIID** Sanfilippo D		N-acetylglucosamine-6-sulfatase	

Musculoskeletal (MSK) abnormalities (including bone and joint) are common across all MPS, and range from joint contractures to deforming abnormalities of the extremities and spine [5]. MSK abnormalities commonly present early in the course of disease and in some cases may be the only symptoms prompting referral [6]. Though MSK symptoms such as stiffness and joint contractures have been noted in small series and case reports, MSK abnormalities in MPS have yet to be evaluated by an objective, validated tool.

The pGALS (paediatric Gait, Arms, Legs, and Spine) is a simple, quick MSK assessment tool validated in school-age children to detect abnormal joints [7]. pGALS was originally developed as a basic approach for non-specialists and adapted from the original Gait, Arms, Legs, and Spine (GALS) examination for adult patients [8]. pGALS consists of three questions about pain and function, followed by a series of simple manoeuvres to assess all major joints to discern normal from abnormal (http://www.arthritisresearchuk.org/health-professionals-and-students/video-resources/pgals.aspx). Abnormalities on pGALS can be followed through with a more detailed regional joint examination and a consensus approach is available [9]. The aim of this study was to describe the use of pGALS in a group of children with established MPS and to assess MSK abnormalities observed.

## Methods

Videos of children with a spectrum of MPS performing pGALS were made at a specialist MPS centre in the UK, by a specialist physiotherapist (PH) as part of their routine care prior to, and independent of, the current study. Informed consent for the use of the videos for teaching and research was obtained. Anonymised patient data (age at the time of the video recording, gender, MPS subtype, history of ERT or HSCT) were made available to the research team. Although historically the terms MPS type I Hurler-Scheie and Scheie syndrome have been used to describe children with more attenuated features of MPS type I, there are no specific criteria for classification of these MPS forms which are regarded as a spectrum of phenotypes. For the purpose of this study, children with MPS type I Hurler-Scheie or Scheie were classified as attenuated MPS type I.

A piloted proforma included the 25 manoeuvres that make up pGALS. The proforma was piloted with videos of two children performing pGALS (one child with juvenile idiopathic arthritis and another with MPS) by four observers (MC, ES, EH and HF) to achieve consensus with definitions for normal/abnormal/not assessable for each of the 25 pGALS manoeuvres. A manoeuvre was considered “not assessable” if it was not performed completely or not clearly observed on video footage. Specific comment on adequate exposure and completion of the manoeuvres was available in a column designated for free text comments. The final proforma was used to assess each video independently by three observers (MC, ES, EH), who were blinded to the MPS subtype. The observers included a paediatric rheumatologist (MC), a paediatric rheumatology trainee (ES) and a specialist paediatric rheumatology physiotherapist (EH). One observer (MC) scored five videos twice on two separate occasions one week apart.

Data were pooled and analysed using descriptive statistics. Percentage scores for the number of abnormal manoeuvres were calculated taking into account three separate scores per manoeuvre per child. The number of abnormal observations per manoeuvre served as a numerator and the total number of committed observations per manoeuvre as denominator (manoeuvres considered “not assessable” were not included in the denominator). Thus, if all four children with MPS type II were noted to have abnormal shoulder flexion, this would mean all three observers noted the same abnormality for all four children i.e. 12 out of 12 scores (3 per each of 4 children) were abnormal. Median ReCal2 programming [10] was used to calculate Kappa scores for inter- and intra-observer consistency.

## Results and discussion

Eighteen videos of children with MPS were assessed by three observers, i.e., a total of 54 sets of ratings and a maximum of 54x25 = 1350 potential scores; 1203 assessable manoeuvres scored. The patient group included 12 boys and six girls with a median age of 11 years at the time of the video recording (range 4–19 years). MPS subtypes included: 13 MPS type I (five MPS type I Hurler, eight MPS attenuated type I); four MPS type II; and one child with mannosidosis (see Table 2). All children with MPS II had attenuated disease without progressive central nervous system involvement. As such, although their IQ was at the lower end of normal, or considered to have a mild intellectual disability, they were able to follow the instructions as deemed by their attempts at the manoeuvres fairly well. Fourteen children had received ERT, of which one had also received an HSCT. Four children had not received any therapy (three MPS type II, one MPS type I).

**Table 2:** Musculoskeletal abnormalities detected by pGALS (number of abnormal observations to total observations)

**Manoeuvre tested**	**pGALS instruction**	**All MPS (n = 18 patients) 1203 total observations**	**MPS I (n = 13)**	**MPS II (n = 4)**	**Mannosidosis (n = 1)**
***Shoulder abduction, external rotation***	*Hands behind neck*	49/54 (91%)	35/39 (90%)	12/12 (100%)	2/3 (67%)
***Shoulder flexion***	*Reach arms up*	48/54 (89%)	36/39 (93%)	12/12 (100%)	0/3 (0%)
***Wrist flexion***	*Hands together back to back*	41/54 (76%)	27/39 (69%)	12/12 (100%)	2/3 (67%)
**Elbow extension**	Reach up	39/53 (74%)	26/39 (90%)	12/12 (100%)	1/2 (50%)
**TMJ excursion**	Open mouth wide and try to put 3 fingers inside	39/54 (72%)	28/39 (72%)	10/12 (83%)	1/3 (33%)
**MCP, DIP, PIP extension**	Hands and wrists together	37/54 (69%)	24/39 (62%)	11/12 (92%)	2/3 (67%)
**Forward flexion of spine**	Bend forwards. Observe curvature of spine from all sides	31/50 (62%)	21/35 (60%)	8/12 (67%)	2/3 (67%)
**Spinal deformity**	Observe patient standing and then bending forwards	28/45 (62%)	22/34 (65%)	5/9 (56%)	1/2 (50%)
**Gait**	Observe patient walking	28/45 (62%)	20/32 (63%)	7/10 (70%)	1/3 (33%)
**Wrist extension**	Hands palm to palm	33/53 (60%)	22/39 (56%)	11/11 (100%)	0/3 (0%)
**MCP/DIP/PIP extension**	Hands out in front	29/52 (56%)	17/37 (46%)	12/12 (100%)	0/3 (0%)
**Cervical spine lateral flexion**	Touch ear to shoulder	29/53 (55%)	17/38 (45%)	12/12 (100%)	0/3 (0%)
**Elbow extension**	Hands out in front	22/40 (55%)	12/27 (44%)	9/11 (82%)	1/2 (50%)
**Ankle dorsiflexion**	Walk on heels	24/45 (53%)	15/33 (45%)	7/9 (78%)	2/3 (67%)
**Neck extension**	Look up to the ceiling	28/53 (53%)	19/38 (50%)	9/12 (75%)	0/3 (0%)
**Forearm supination**	Turn hand over	20/42 (48%)	10/30 (33%)	9/11 (82%)	1/1 (100%)
**Opposition of thumb and 3**^**rd**^**-5**^**th **^**fingers**	Touch tips of fingers with thumb	23/54 (43%)	14/39 (36%)	8/12 (67%)	1/3 (33%)
**MCP/DIP/PIP flexion**	Make a fist	20/54 (37%)	10/39 (26%)	10/12 (83%)	0/3 (0%)
**Elbow flexion**	Put hands behind neck	12/42 (29%)	6/27 (33%)	5/12 (42%)	1/3 (33%)
**Knee extension**	Bring ankle up to bottom	12/44 (27%)	7/29 (24%)	4/12 (33%)	1/3 (33%)
**Knee flexion**		11/45 (24%)	4/30 (13%)	7/12 (58%)	0/3 (0%)
**Opposition of thumb and index finger**	Touch tip of finger with thumb	10/54 (19%)	5/39 (13%)	5/12 (42%)	0/3 (0%)
**Elbow flexion**	Put hands and wrists together	7/53 (13%)	5/39 (13%)	2/11 (18%)	0/3 (0%)
**Ankle plantar flexion**	Walk on tip-toes	4/45 (9%)	2/33 (6%)	2/9 (22%)	0/3 (0%)

**Key:** DIP = distal interphalangeal; MCP = metacarpophalangeal; PIP = proximal interphalangeal; pGALS = paediatric Gait, Arms, Legs and Spine; TMJ = temporomandibular joint.

Table 2 summarises the abnormalities detected using pGALS for the whole patient group and then according to MPS subtypes. In the majority, all the manoeuvres were scored by all observers, although hip manoeuvres were difficult to assess as some children were not undressed or the views were obscured. For the whole group of children, restriction of joint movement was frequently seen and in order of descending frequency, the most commonly affected joints were in the upper limb (shoulders, wrists, elbows, fingers), temporomandibular joints (TMJs), neck and lower limbs (ankles and knees). Hip evaluation as per pGALS maneouvre was not performed in the videos or the video footage did not include adequate views to assess hip movement. Gait was abnormal in many children with free text comments made about toe walking, slow walking, or unstable gait. At each joint, specific movements were more commonly abnormal than others – for example at the shoulder (forward flexion and abduction/external rotation (90%)), elbow (extension 72%), wrist (flexion 74%), fingers (extension 67%), temporomandibular joint excursion (70%), spine (forward flexion 62%), and ankles (dorsiflexion 52%). Spinal deformity was visible in 61%.

All components of the pGALS manoeuvres demonstrated abnormalities. The three manoeuvres most likely to demonstrate joint abnormalities were, “Raise your hands straight in the air to the sky”, “Put your hands together back to back”, “Put your hands behind your neck”, (See Table 2, italicized text). Observing gait, spine and forward spinal flexion were also informative. Some children (17%, n = 3) had difficulty with walking and especially walking on their heels or toes and two (11%) had genu valgum. Free text comments included observations of short stature (17%, n = 3), wearing glasses (45%, n = 8), hearing aids (17%, n = 3), dysmorphism (89%, n = 16), dental abnormalities (6%, n = 1), and short, thickened fingers (28%, n = 5).

Patterns of joint abnormalities were analysed by MPS subtype (Tables 3, 4 and 5). With respect to the different MPS subtypes, the same pattern of joint involvement was apparent using pGALS and in decreasing order of frequency, restriction of shoulders, wrists, elbows, fingers, TMJs and spine were most common. Gait and spinal appearance were also commonly abnormal. These abnormalities were more frequent in MPS II compared to the MPS I. Dysmorphism was observed in all MPS type I Hurler and II patients and noted to be mild in the majority [n = 7, 88%] of the MPS attenuated type I patients. Manoeuvre-specific difficulties arose with asking children to put three fingers into their mouths, and asking children to make a fist, both of which required repeat instructions in some patients. Physically difficult manoeuvres for some children included walking and bending over (spinal flexion), which children would attempt but be unable to perform completely or perform very slowly. This was more apparent in those with pronounced contractures and skeletal abnormality.

**Table 3:** Musculoskeletal abnormalities detected by pGALS in MPS type I (Hurler)

**Manoeuvre tested**	**pGALS instruction**	**MPS I (Hurler) (n = 5)**
**Spinal deformity**		15/15 (100%)
**Shoulder flexion**	Reach arms up	13/15 (87%)
**TMJ excursion**	Open mouth wide and try to put 3 fingers inside	13/15 (87%)
**Forward flexion of spine**	Bend forwards. Observe curvature of spine from all sides	11/15 (73%)
**Shoulder abduction, external rotation**	Hands behind neck	11/15 (73%)
**Wrist flexion**	Hands together back to back	10/15 (67%)
**Elbow extension**	Reach up	9/15 (60%)
**Gait**	Observe patient walking	9/15 (60%)
**MCP, DIP, PIP extension**	Hands and wrists together	8/15 (53%)
**Neck extension**	Look up to the ceiling	7/15 (47%)
**Wrist extension**	Hands palm to palm	6/15 (40%)
**MCP/DIP/PIP extension**	Hands out in front	6/15 (40%)
**Ankle dorsiflexion**	Walk on heels	6/15 (40%)
**Elbow extension**	Hands out in front	4/11 (36%)
**Cervical spine lateral flexion**	Touch ear to shoulder	5/15 (33%)
**Forearm supination**	Turn hand over	4/12 (33%)
**Opposition of thumb and 3**^**rd**^**-5**^**th **^**fingers**	Touch tips of fingers with thumb	4/15 (27%)
**Knee extension**	Bring ankle up to bottom	2/14 (14%)
**Knee flexion**		1/15 (7%)
**Elbow flexion**	Put hands behind neck	1/15 (7%)
**Elbow flexion**	Put hands and wrists together	1/15 (7%)
**Ankle plantar flexion**	Walk on tip-toes	0/15 (0%)
**MCP/DIP/PIP flexion**	Make a fist	0/15 (0%)
**Opposition of thumb and index finger**	Touch tip of finger with thumb	0/15 (0%)

Figures stated as number of abnormal observations to number of total observations.

**Table 4:** Musculoskeletal abnormalities detected by pGALS in MPS attenuated type I

**Manoeuvre tested**	**pGALS instruction**	**MPS Ia (n = 8)**
**Shoulder abduction, external rotation**	Hands behind neck	24/24 (100%)
**Shoulder flexion**	Reach arms up	23/24 (96%)
**Elbow extension**	Reach up	17/24 (71%)
**Wrist flexion**	Hands together back to back	17/24 (71%)
**Wrist extension**	Hands palm to palm	16/24 (67%)
**MCP, DIP, PIP extension**	Hands and wrists together	16/24 (67%)
**Gait**	Observe patient walking	11/17 (65%)
**TMJ excursion**	Open mouth wide and try to put 3 fingers inside	15/24 (62%)
**Spinal deformity**		7/19 (56%)
**Forward flexion of spine**	Bend forwards. Observe curvature of spine from all sides	10/19 (53%)
**Neck extension**	Look up to the ceiling	12/23 (52%)
**Cervical spine lateral flexion**	Touch ear to shoulder	12/23 (52%)
**MCP/DIP/PIP extension**	Hands out in front	11/22 (50%)
**Elbow extension**	Hands out in front	8/16 (50%)
**Ankle dorsiflexion**	Walk on heels	9/18 (50%)
**MCP/DIP/PIP flexion**	Make a fist	10/24 (42%)
**Opposition of thumb and 3**^**rd**^**-5**^**th **^**fingers**	Touch tips of fingers with thumb	10/24 (42%)
**Elbow flexion**	Put hands behind neck	5/12 (42%)
**Forearm supination**	Turn hand over	6/18 (33%)
**Knee extension**	Bring ankle up to bottom	5/15 (33%)
**Knee flexion**		3/15 (20%)
**Opposition of thumb and index finger**	Touch tip of finger with thumb	5/24 (21%)
**Elbow flexion**	Put hands and wrists together	4/24 (17%)
**Ankle plantar flexion**	Walk on tip-toes	2/18 (11%)

**Table 5:** Musculoskeletal abnormalities detected by pGALS in MPS type II

**Manoeuvre tested**	**pGALS instruction**	**MPS II (n = 4)**
**Shoulder flexion**	Reach arms up	12/12 (100%)
**Shoulder abduction, external rotation**	Hands behind neck	12/12 (100%)
**Elbow extension**	Reach up	12/12 (100%)
**Wrist flexion**	Hands together back to back	12/12 (100%)
**Wrist extension**	Hands palm to palm	11/11 (100%)
**MCP/DIP/PIP extension**	Hands out in front	12/12 (100%)
**Cervical spine lateral flexion**	Touch ear to shoulder	12/12 (100%)
**MCP, DIP, PIP extension**	Hands and wrists together	11/12 (92%)
**TMJ excursion**	Open mouth wide and try to put 3 fingers inside	10/12 (83%)
**MCP/DIP/PIP flexion**	Make a fist	10/12 (83%)
**Elbow extension**	Hands out in front	9/11 (82%)
**Forearm supination**	Turn hand over	9/11 (82%)
**Ankle dorsiflexion**	Walk on heels	7/9 (78%)
**Neck extension**	Look up to the ceiling	9/12 (75%)
**Gait**	Observe patient walking	7/10 (70%)
**Forward flexion of spine**	Bend forwards. Observe curvature of spine from all sides.	8/12 (67%)
**Opposition of thumb and 3**^**rd**^**-5**^**th **^**fingers**	Touch tips of fingers with thumb	8/12 (67%)
**Spinal deformity**		5/9 (56%)
**Opposition of thumb and index finger**	Touch tip of finger with thumb	5/12 (42%)
**Elbow flexion**	Put hands behind neck	5/12 (42%)
**Knee extension**	Bring ankle up to bottom	4/12 (33%)
**Knee flexion**		7/12 (58%)
**Ankle plantar flexion**	Walk on tip-toes	2/9 (22%)
**Elbow flexion**	Put hands and wrists together	2/11 (18%)

**Figure Key**: ANA = antinuclear antibody; ESR = erythrocyte sedimentation rate; IDUA = iduronidase, alpha-L; JIA = juvenile idiopathic arthritis; NSAIDs = non-steroidal anti-inflammatory drugs; pGALS = paediatric Gait, Arms, Legs and Spine; RA = rheumatoid arthritis; uGAG = urinary glycosaminoglycans.

Abnormalities on pGALS varied with age. Younger children ages 0–10 years (n = 9, 27 observations), in comparison to older children ages 11–19 (n = 9, 27 observations) were observed to have fewer dysmorphic features (52% [12/23] younger versus 80% [20/25] older), restriction of shoulder flexion and abduction (83% [20/24] and 85% [23/27] versus 96% [26/27] and 100% [27/27]), TMJ excursion (67% [18/27] versus 81% [22/27]), MCP extension (73% [19/26] versus 85% [22/26]) and spinal deformities (54% [14/26] versus 68% [13/19]). All other manoeuvres yielded <50% abnormal scores. All but one of these children had received ERT treatment. Children ages 11–19 were all noted to be dysmorphic. Seven children had received ERT, with one having also received an HSCT.

Consistency between observers was very good with a mean intra-observer Kappa 0.74 (range 0.65-0.88) and a mean inter-observer Kappa 0.62 (range 0.51-0.77). No apparent discordance in agreement was noted.

This is the first study to describe MSK abnormalities in children with MPS using pGALS as a standardized and validated simple MSK examination tool. We demonstrate that pGALS performs well to identify abnormal joints with joint restriction with the upper limbs, TMJ, neck and spine being the most commonly observed pattern of joint involvement. We demonstrate that pGALS is able to discern a consistent pattern of joint involvement across various MPS subtypes with changes being more marked in MPS type II compared to the attenuated MPS type I. We observed that pGALS also performs well in attenuated MPS type I, including younger children. This is important since these children often present with MSK features, invariably subtle and in isolation from other system involvement, resulting in significant delay in diagnosis [11].

The MSK abnormalities observed using pGALS are similar to those cited in the literature with marked and widespread joint restriction with preferential involvement of the upper limbs, most notably the shoulders [12] and interphalangeal joints causing fixed flexion deformities in the fingers [5,6]. Our patient group had a large spectrum of ages and in the younger children with the attenuated subtype, predominant involvement of the upper limb was observed. We cannot comment fully on lower limb contractures as hip involvement was not adequately assessed. Limited TMJ excursion was common and may reflect true joint damage, the presence of “bullet phalanges” (large, round fingers observed in some children with MPS), or oral crowding as a result of macroglossia or dental abnormalities [13].

There are several limitations to our study. Firstly, the number of children included was relatively small and did not represent all subtypes of MPS. Given the rarity of MPS, however, we consider the study to present clinically important findings. Secondly, this was a retrospective assessment of videos recorded as part of routine clinical assessment at a specialist MPS centre in the UK; the available videos did not provide adequate views of hip assessment in some cases and the videos had been edited. Consequently it was not clear how many attempts were needed, whether the instructions to the children were fully understood and we were not able to evaluate the time taken to perform manoeuvres. There is, therefore, a need to validate our findings prospectively in an additional cohort of children, particularly with respect to hip assessment. Thirdly, the group of children were not incident cases and many had established disease. It is therefore not possible to comment on the utility of pGALS in detecting previously undiagnosed disease. It was, however, able to discern abnormal joints even in the younger children with milder forms of MPS and in particular the attenuated phenotype.

**Figure 1:**
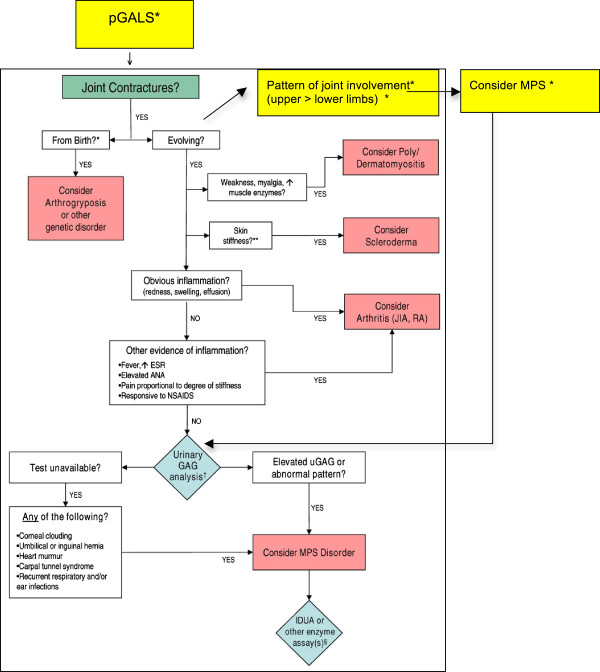
Suggested Revisions (highlighted yellow with asterisk*) to Existing Algorithm for Evaluation of Joint Contractures in Children. Adapted from Cimaz et al. [17].

The pGALS examination was originally developed in the context of paediatric rheumatology clinics but has been shown to be useful to detect pathology other than rheumatic disease in acute general paediatric assessment [14,15]. Furthermore pGALS has been shown to detect joint abnormalities that may not be apparent from history alone [16]. Our study suggests that pGALS may be a useful adjunct to facilitate earlier recognition of MPS and this is of clinical importance as a large number of children in this study had attenuated MPS type 1. Such patients often present with subtle contractures and delays in diagnosis [11,17]. Given the availability of ERT, this delay is concerning as early intervention is likely to improve the clinical outcomes for these children. It is increasingly important to develop strategies to facilitate early diagnosis and the algorithmic approach to evaluation of the child with joint contractures is welcome [17] [See Figure 1].

## Conclusions

Our study suggests that pGALS may be an effective tool to detect MSK changes, including joint contractures and the characteristic pattern of joint involvement in MPS. Further work is needed to validate our findings and develop educational strategies to facilitate recognition of MPS, access to specialist care and ultimately improve clinical outcomes.

## Abbreviations

DIP: Distal interphalangeal; ERT: Enzyme replacement therapy; HSCT: Haematopoietic stem cell transplantation; MCP: Metacarpophalangeal; MPS: Mucopolysaccharidoses; MSK: Musculoskeletal; PIP: Proximal interphalangeal; pGALS: Paediatric Gait, Arms, Legs and Spine; TMJs: Temporomandibular joints.

## Competing interests

With regards to conflicts of interest, Professor Helen Foster has received honoraria and educational bursaries from Genzyme and BioMarin. Neither of these pharmaceutical companies were involved in the study; collection, analysis and interpretation of data; writing of the report; or the decision to submit the paper for publication. No honoraria, grants or any other forms of payment were given to produce the manuscript.

## Authors’ contributions

Contributors’ statements are as follows: MOC: designed the data collection instrument; collected, collated and analysed the data; drafted the initial manuscript; reviewed and revised the manuscript; and approved the final manuscript as submitted. ESS: designed the data collection instrument; collected and analysed data; reviewed and revised the manuscript, and approved the final manuscript as submitted. EH: designed the data collection instrument, collected data, reviewed and revised the manuscript, and approved the final manuscript as submitted. PH: performed and filmed the videos from which data for our study was collected. She also reviewed and revised the manuscript, and approved the final manuscript as submitted. EW: advised on the drafting of the manuscript. Sadly, Dr. Wraith passed away suddenly during the drafting of this manuscript and as such, was unable to approve the final manuscript as submitted. SJ: reviewed and revised the manuscript, and approved the final manuscript as submitted. TR: advised on the conceptualization and design of the study, data collection instruments, and data analysis. He also reviewed and revised the manuscript, and approved the final manuscript as submitted. HEF: conceptualized the study design; coordinated and supervised design of data collection instruments, data collection, and analysis. Dr. Foster also reviewed and revised the manuscript, and approved the final manuscript as submitted. All authors read and approved the final manuscript.
